# Ways of Being Together During the COVID-19 Pandemic: Support Bubbles and the Legal Construction of Relationships

**DOI:** 10.3389/fsoc.2021.730216

**Published:** 2021-09-02

**Authors:** Sarah Trotter

**Affiliations:** Department of Law, London School of Economics and Political Science, London, United Kingdom

**Keywords:** dating, extended households, family law, friendship, interventions in family life, support bubbles, the nuclear family

## Abstract

This article focuses on the concept of the support bubble. The concept was introduced in New Zealand in March 2020 in the context of the COVID-19 pandemic to denote a network of people with whom a person could have physical contact, and was later taken up in various forms elsewhere, particularly in the UK. The article focuses on the meaning that was attached to the concept and to the ways of being together that it encapsulated and stipulated. Where support bubbles were formalised as a matter of law, as in New Zealand and the UK, a particular form of relating was legally constructed and real relationships were affected through law. The article addresses the meaning and implications of the concept of the support bubble in this light. First, it considers the concept of the support bubble as a new legal form, which drew in, and built on, a range of relationships and then recast them in terms of a new legal form. Second, it analyses the central question posed by the concept as one of the meaning of being together in a support bubble, not only for those navigating and living with the concept in practice, but also as mediated in and through law. Third, it outlines how the concept of the support bubble represented a distinct legal development. It enabled those who were eligible to define for themselves, albeit within a specified framework, the meaning and nature of a relationship of support of this kind. It also supplied a space in which some kinds of relationships that had not necessarily attracted much previous legal attention—like friendships and dating relationships—came to find a degree of legal reflection and recognition.

## Introduction

Before the onset of the COVID-19 pandemic, and the disruption that it unleashed on ways of thinking about, relating to, and being with one another and ourselves (Kristeva, 2021), there was no notion of a support bubble. The concept that later emerged was a product of a previously unthinkable reality: a COVID-19 concept, introduced in New Zealand in March 2020, and later taken up in various forms in other countries to denote “an exclusive social unit whose members are allowed physical contact amongst themselves but not with others” (Trnka and Davies, 2021, p. 167). The aim, according to Tristram Ingham (2020), who came up with the idea of the bubble, was to capture the containment and protection required during the pandemic, and to do so in an empowering way. A bubble was an apt metaphor in that respect, being a “fragile yet beautiful structure that has to be nurtured and preserved” (Ingham, 2020). It reflected, in multiple and complex ways, the essence of two basic imperatives that structured life in this period: of being together apart and being apart together.

This article focuses on the form that these ways of being together took in the context of the support bubble. In particular, it explores the meaning that was attached to the concept and, therefore, to the ways of being together that it encapsulated and stipulated. Where support bubbles were formalised as a matter of law, as in New Zealand and the United Kingdom, the question of meaning was in part a legal one. In these cases, a form of relating was legally constructed, and real relationships were affected through law. What was especially notable from a legal perspective was the range of relationships that were implicated. In New Zealand, for example, a bubble was initially confined to a single household, with a few limited exceptions. The concept of the bubble accordingly drew together a diverse range of household relationships between family members, friends, and relative strangers living together. In so doing, it simultaneously–and inevitably–affected cross-household relationships. This effect was furthered when the possibility of expanding household bubbles was introduced and “multi-household bubbles” emerged as a relational form in New Zealand. This model subsequently influenced the versions of the support bubble that were eventually adopted across the United Kingdom, which similarly implicated a wide range of relationships. The primary aim was to enable the (re)connection of those living alone (or parenting alone) with certain family members, friends, relatives, or loved ones.

To speak of relationships in terms of their “kind” is not without problems; it is a reductive mode of expression that overlooks the particularity of a given relationship and subjects it to a specific form. However, relationships are categorised according to their “kind” in law, and the concept of the support bubble raises three issues about the kinds of relationships that it drew together. Firstly, kinds of relationship that had not necessarily attracted much previous legal attention–like friendships and dating relationships–found a space in law by being accorded a degree of legal reflection and recognition. Secondly, the concept of the bubble was built around the unit of the household and was accordingly shaped by normative assumptions that were made about households and relationships within and across households (Gulland, 2020; Long, 2020; Trnka and Davies, 2021). Thirdly, the relationships that were encapsulated and enabled by the concept of the support bubble would not ordinarily have been categorised together or treated as comparable in law. The legal concept of the support bubble was distinctive in drawing together different household and cross-household relationships. It then recast them in terms of a new legal kind: the supportive kind. The support bubble thus presented not only as “a new social form” (Long et al., 2020, p. 55), but also as a new legal form.

This reduction of relationships to a legal form that was, simultaneously, constructed as a new way of relating raises a number of questions about the structure of the concept of the support bubble itself. They include questions about the conceptualisation of support-bubble relationships, the assumptions that were made in this context, particularly as to eligibility and capacity to act on eligibility, and the potential implications of the concept for the legal recognition and regulation of close relationships beyond the COVID-19 pandemic. The concept of the support bubble also raises more fundamental questions about the socio-legal construction of support in this context and about the meaning ascribed to it. These questions are explored in this article.

The first part traces the introduction of the concept of the support bubble in New Zealand, where bubbles were included from the outset as part of the lockdown plan. The second part outlines the spread of the concept to the United Kingdom. The purpose in doing so is not a comparative analysis per se; the policies and epidemiological situations were not directly comparable (Scientific Pandemic Insights Group on Behaviours, 2020, p. 1). But the United Kingdom was most notably influenced by New Zealand’s bubble strategy (Han et al., 2020, p. 1527; HM Government, 2020a), and so the article considers how England, Scotland, Wales, and Northern Ireland each constructed a version of the concept as part of their lockdown exit strategies. The third part addresses how, in both New Zealand and the United Kingdom, the concept of the support bubble became a new socio-legal construct to be navigated. It considers how the concept encapsulated and specified certain ways of being together and the meaning that was attached to them. Finally, the article reflects on the implications of the legal construction of support-bubble relationships in the context of broader debates about the types of close relationships that are recognised in and regulated by law.

## The Introduction of the Concept of the Support Bubble

The concept of the support bubble was developed in New Zealand. It was introduced as part of the four-level COVID-19 Alert Level System announced on March 21, 2020 (New Zealand Government, 2021b). On 25 March, and following a 48-h notice period, New Zealand moved to the highest alert level (Alert Level 4), entailing a nationwide lockdown with “the entire nation [going] into self-isolation” (New Zealand Government, 2021d). People “outside essential services” were told to “stay at home, and to stop all interactions with others outside of those in [their] household” (Ardern, 2020a). The introduction of the concept of the bubble followed almost immediately, with residents being told the next day that they needed to “stick to [their] bubble”, whatever it was, for the duration of the period of self-isolation (Devlin and Manch, 2020). 9 days later, on 3 April, this instruction was formalised in an isolation order issued by the Director-General of Health under Section 70 (1) (f) of the Health Act 1956. In a later challenge to the legality of the original instruction to stay at home, the High Court of New Zealand (New Zealand High Court, 2020) held that the messages to do so from 26 March–3 April had in fact unlawfully limited certain rights and freedoms under the New Zealand Bill of Rights Act 1990: namely the rights to freedom of movement, peaceful assembly, and association. The Court held that, although the effect of the stay-at-home requirement had been to limit these rights, the requirement itself had not been prescribed by law.

The isolation order of April 3, 2020 essentially required “all persons within all districts of New Zealand to be isolated or quarantined”, and, in particular, “to remain at their current place of residence, except as permitted for essential personal movement; and … to maintain physical distancing … ” [Section 70 (1) (f) of the Health Act 1956]. One of the categories of permission for “essential personal movement” was “shared bubble arrangements”. This seems to be the first appearance in law of the concept of the bubble itself. Under this category, and where a “shared bubble arrangement” was in place, a child could visit and stay with “another joint care-giver”; and a person could visit or stay at another residence if “[one] person lives alone in [one], or both, of those residences; or all persons in one of those residences are vulnerable”. It was also possible for a person to leave their residence to “assist a fellow resident to travel to or from” one of those residences.

These exceptions–and the very notion of the “shared bubble”—had been made clear from the outset of the Level 4 period. Permission had been granted to those living alone to “buddy up” with another person living alone locally (New Zealand Herald, 2020). Those needing help with childcare, such as essential workers, were advised to “identify a trusted buddy–as long as they’re not elderly or vulnerable in other ways”, who could “become the child’s caregiver” (New Zealand Government, 2021c, p. 5). Families living apart, such as separated couples with shared care of their children, were allowed to form a single bubble (Ardern, 2020b, p. 6).

Although the concept of the “bubble” was largely household-focused, in that people were instructed to conceive of anyone with whom they lived as constituting their bubble, the two terms were not entirely synonymous. For some people, support from beyond the household would be necessary (New Zealand Government, 2021c, p. 5), and emphasis was instead placed on keeping “whatever your bubble is for the month” as small and tight as possible (Ardern, 2020b, p. 6; New Zealand Government, 2021c, p. 5). At the post-Cabinet press conference on March 24, 2020, the prime minister, Jacinda Ardern, was asked by a member of the press about the way in which the “self-isolation regime” seemed to be “heavily geared towards households”. In replying to this question–and articulating the possibility of cross-household bubbles for co-parents–Ardern stated that what she was really asking people to do was “to just apply common sense and common principles” (Ardern, 2020b, p. 6). The critical point was the principle of exclusivity: people were to remain within and maintain their bubble once it had been established, and to keep their distance from anyone outside it. In a later study of the characteristics of Level 4 bubbles, it was found that most bubbles formed by survey respondents were small, containing three to four people, and that 80% contained a single household (Kearns et al., 2021).

The basic principle of the exclusivity of the bubble was reiterated when New Zealand moved to Alert Level 3 on April 27, 2020 and “extended” bubbles became possible. At this point, residents were still legally required to remain within their bubbles “whenever [they were] not at work or school”, but they were permitted to expand their bubble to “connect with close family and whānau [an extended family or community of related families], bring in caregivers, or support isolated people” (New Zealand Government, 2021a). These “extended bubble arrangements and shared caregiving arrangements” were permitted as “essential personal movement” under Section 7 of the Health Act (COVID-19 Alert Level 3) Order 2020. In 2020, 47.6% of the respondents to a survey on New Zealanders’ experiences of lockdown were found to have expanded their bubble in this way (Long et al., 2020, p. 28).

## The Spread of the Concept of the Support Bubble

Other countries, and particularly the United Kingdom, began looking to the concept of the bubble as part of lockdown easing strategies (Drakeford, 2020a; HM Government, 2020a). The aim was to permit increased contact, particularly for those identified as having the greatest need, while limiting the epidemic risk involved (Block et al., 2020; Leng et al., 2021). Bubbles were presented as a way of alleviating some of the worst effects of the loneliness, isolation, and separation involved in the original lockdowns. In Belgium, for example, which went into national lockdown on March 18, 2020, social bubbles were introduced to coincide with Mother’s Day in May 2020. In announcing the plan, Sophie Wilmès, the prime minister, stated: “The physical separation from those whom we love has in some cases become unbearable…” (Rankin, 2020). This version of the bubble permitted households to invite up to four “guests” to their home, although they were expected to remain at a 1.5 m distance from one another.

The United Kingdom similarly introduced support bubbles as part of a broader lockdown easing strategy in 2020: from 13 June in England and Northern Ireland, 19 June in Scotland, and 6 July in Wales. The main target, at least initially, was individuals living alone. Whereas New Zealand had always had a lockdown “buddy” system for people living alone, the United Kingdom had not. As Boris Johnson (2020a), the United Kingdom Prime Minister, stated in issuing the instruction to “stay at home” on 23 March: “[y]ou should not be meeting friends. If your friends ask you to meet, you should say No. You should not be meeting family members who do not live in your home”.

In further guidance issued that same day, it was made clear that “[w]here parents … do not live in the same household, children under 18 can be moved between their parents’ homes to continue existing arrangements for access and contact” (HM Government, 2020b, Section 1). This guidance was subsequently set out in the corresponding regulations in the four United Kingdom nations, and advice on what it meant in practice was issued by Sir Andrew McFarlane (2020), President of the Family Division of the High Court (England and Wales). But exceptions to the stay-at-home requirement were otherwise limited, and there was no generalised provision for those living alone comparable to that which existed in New Zealand. The effect was that many people living alone in the United Kingdom did not have any permissible way of actually being with another person from March 23, 2020 until restrictions began to be eased later that spring. How this easing occurred varied across the four United Kingdom nations, which each had their own regulations and restrictions.

Three broad stages can be identified in the elaboration of the concept of the support bubble across the United Kingdom. First came the possibility of meeting people from other households outdoors. From May 13, 2020, people living in England were allowed to meet one person from another household in “a public open space for the purposes of open-air recreation to promote their physical or mental health or emotional wellbeing” [The Health Protection (Coronavirus, Restrictions) (England) (Amendment) (No. 2) Regulations 2020, r (2) (3) (a) (iii)]. In Northern Ireland, from May 19, 2020, groups of up to six people from different households were allowed to meet outdoors (The Executive Office, 2020a). In Scotland, from May 29, 2020, groups of up to eight people from two households were allowed to meet outdoors. In all cases, people were advised to follow social-distancing guidelines and to exercise caution. For example, in Scotland, people were told that they should not meet more than one household at a time, or more than one household per day; that they should not “share items” or “touch the same surfaces as another household”, meaning that households meeting for a picnic or barbeque needed to bring their own “food, cutlery, plates, or cups”; and that they should not go indoors when meeting another household (Sturgeon, 2020a). The stated aim was to enable family and friends to see each other while mitigating the risk involved.

It was in this spirit that people living in England and Wales were similarly, and finally, allowed to meet in small groups outdoors (including in private gardens) from June 1, 2020. In Wales, two households were permitted to meet, although the requirement that people remain within their local area (within five miles of their home) meant that “this [did] not allow people with loved ones outside their local area to meet, unless they are providing care to a vulnerable person” (Drakeford, 2020c). In England, up to six people were allowed to meet (Johnson 2020b) [The Health Protection (Coronavirus, Restrictions) (England) (Amendment) (No. 3) Regulations 2020, r2 (7)]. The Prime Minister (Johnson, 2020b) explained that “friends and family [could] start to meet their loved ones–perhaps seeing both parents at once, or both grandparents at once”: a moment that would be “for many … a long-awaited and joyful” occasion.

Against this backdrop came the second stage in the elaboration of the concept of the support bubble across the United Kingdom: the introduction of the concept itself. On 10 June 2020, Johnson (2020c) announced that from 13 June, people in England who were living alone or in a single-parent household (with children under 18) would be able to form a “support bubble” with one other household. The aim was “to support those who [were] particularly lonely as a result of lockdown measures” and “to limit the most harmful effects of the … social restrictions”, bearing in mind that despite the earlier relaxation of the rules on meeting outdoors, “there [were] still too many people, particularly those who live by themselves, who [were] lonely and struggling with being unable to see friends and family”. Those in a bubble “[would] be able to act as if they [lived] in the same household”, but bubbles had to be exclusive. The regulations were amended accordingly, and support bubbles were introduced into English law as “linked households”, although the term that was used in practice and in the guidance from the Department of Health and Social Care was “support bubble” (Department of Health and Social Care, 2021b).

In the days and weeks that followed, Northern Ireland, Scotland, and Wales similarly introduced versions of the concept of the support bubble. In Northern Ireland, First Minister Arlene Foster announced on June 11, 2020 that “indoor visits with one other household” would be permitted for those living alone from 13 June–a “further piece”, Foster said, of “the normalisation jigsaw, as we emerge from lockdown” (The Executive Office, 2020b). In Scotland, from June 19, 2020 people living alone or living only with children under the age of 18 were allowed to form an exclusive “extended household group” with another household (Sturgeon, 2020b). In Wales, First Minister Mark Drakeford (2020a) announced on June 29, 2020 that, from 6 July, it would be possible for two separate households to form an exclusive “single extended household”. “The Welsh Government”, Drakeford said, “[had] drawn on experience from around the world where this concept [had] been successfully introduced, including in New Zealand”.

The third stage in the elaboration of the concept of the support bubble across the United Kingdom involved the development of the four different versions over the course of the following year. This occurred in different and complex ways, and the guidance and regulations across the four United Kingdom nations were repeatedly amended. In essence, however, two main types of adjustments were involved: to the eligibility criteria and rules in relation to forming and dissolving bubbles, as in England (Department of Health and Social Care, 2021b), and to the structure and scope of the concept itself, as in Wales (Welsh Government, 2021c; Welsh Government, 2021d).

For example, in England, whereas initially only those living alone, or in a single parent-household, with children under 18, could form a support bubble, eligibility was eventually extended to include households comprised of “one or more children and no adults”; households with a child “under the age of one or [who was] under that age on 2nd December 2020”; households with a child who “[has] a disability, [requires] continuous care and [is] under the age of five, or [was] under that age on 2nd December 2020”; and those who were the only adult in their household not requiring continuous care as a result of a disability [The Health Protection (Coronavirus, Restrictions) (Steps) (England) Regulations 2021, r (3) (2)]. In Scotland, eligibility was extended to those who were “part of a couple who lives apart”; children whose parents were separated could “move freely between both parents’ households” without needing to form an extended household (Scottish Government, 2021a). Other exemptions for the purposes of providing care and support were created; for instance, even under the highest level of restrictions, it was possible to go into another person’s home “to provide care and assistance to a vulnerable person” (Scottish Government, 2021b). In Northern Ireland, it eventually became possible for two households of any size to link and form an exclusive household bubble specifically “for the purpose of the members of either linked household providing care or welfare support to members of the other linked household” [The Health Protection (Coronavirus, Restrictions) Regulations (Northern Ireland) 2021, r19 (2)], but this followed periods in which cross-household mixing was restricted and bubbles were only permitted for single-person households or those with caring responsibilities.

Wales had started out with a wider version of the support bubble, enabling two separate households to form an exclusive “single extended household”. This version was subjected to changes over the following months, and the number of households that could be included in the bubbles fluctuated according to coronavirus case numbers. Under the highest alert level of a four-tier system that was later introduced in December 2020, extended households were suspended entirely, as they had also been during the autumn in areas where local restrictions had been imposed (Welsh Government, 2021a; Welsh Government, 2021b). However, “single people household bubbles” were possible in limited circumstances, including for single parents and those living alone (Drakeford, 2020b). The original version of the support bubble in Wales (the “extended household”) was accordingly fragmented, with the introduction of a narrower version of a support bubble too.

The versions of the support bubble that were constructed in England, Scotland, Wales, and Northern Ireland formed part of far-reaching restrictions in relation to meeting, gathering, and travelling. Complex questions arose, for example, about cross-border bubbles and the regulations that applied in these cases. Parallel concepts were also introduced, such as “childcare bubbles” in England, enabling households with at least one child aged 13 or under to link with another “for the purpose of the second household providing informal childcare” [The Health Protection (Coronavirus, Restrictions) (Steps) (England) Regulations 2021, r4 (2)]; (Department of Health and Social Care, 2021a); “school bubbles”, grouping children and staff according to year-group or class to reduce the risk of transmission (Department for Education, 2021); and “Christmas bubbles” across the United Kingdom, allowing some household mixing over Christmas 2020, other than in parts of England that were under the highest tier four restrictions (BBC, 2020a).

The resulting landscape of the support bubble in the United Kingdom was a complex one. However, the central ideas that underpinned each version of the concept were the notions of containment and support that had motivated the model developed in New Zealand. In both contexts, households, whether singular, or extended or linked, were encouraged to think of themselves as bounded: a move that was underpinned by an unprecedented level of intervention in personal, familial, and social life and demanded a fundamental reconceptualisation of the meaning of being together.

## The Support Bubble as a Way of Relating

The concept of the support bubble raised questions that had to be addressed for the first time: What did it mean to be in a bubble together? What did it mean to relate in this way? Answers to these questions and experiences of bubbles would inevitably differ according to individual circumstances and household composition (Long et al., 2020; Okabe-Miyamoto et al., 2021; Trnka et al., 2021). Bubbles themselves, however, were constructed as involving a form of collective identity, and the dominant principle of the bubble was that of exclusivity. Members of a bubble were “allowed physical contact amongst themselves but not with others” (Trnka and Davies, 2021, p. 167), and the maintenance of a distance between bubbles was treated as an expression of care. This distance was not only expressed in spatial terms, as reflected in the social distancing rules and requirements to maintain a distance from members of other bubbles, but also in temporal terms, as reflected in the periods of time that had to lapse between the dissolution of an old bubble and the formation of a new one. In England, for example, when cross-household support bubbles were introduced, the rule on dissolution was that, if two households “ceased being linked households”, neither could be “linked with any other household” [The Health Protection (Coronavirus, Restrictions) (England) (Amendment) (No. 4) Regulations 2020, r2 (7)]. This stipulation was subsequently dropped to allow for the possibility of changing a bubble, although the guidance stated that this should be avoided “[w]here possible” and that there should be a gap of ten days between the dissolution of one bubble and the formation of another (Department of Health and Social Care, 2021b). Some months later, Europe was more broadly described as “moving towards a new form of coexistence based on household bubbles” (Güell, 2020). But bubbles did not only coexist. They were related to one another, since the distance between them had to be constructed and maintained: the relation between bubbles was one of being apart, but together.

The meaning of being together in a bubble was a question that people had to navigate for the first time. Confinement to household bubbles necessitated a reimagining of everyday life (Appleton, 2020); exceptions to household confinement and the possibility of cross-household bubbles were meanwhile linked to the presumed needs and vulnerabilities of a defined population who were identified according to their living arrangements. In the United Kingdom, cross-household support bubbles were targeted, at least initially, at those living alone, or parenting alone. Wales constituted an exception insofar as the original version of the support bubble, the “single extended household”, was aimed not only at alleviating loneliness and isolation but also at enabling family reunions and supporting families with childcare responsibilities. When the possibility of forming extended households was subsequently restricted, attention shifted to those living alone and parenting alone, and “single people household bubbles” were introduced. Throughout, however, the focus was on need: in introducing extended households, the First Minister of Wales urged people to think about “who needs support and would benefit most from joining an extended household” (Drakeford, 2020d).

At the time of the original introduction of the concept of the support bubble across the United Kingdom (from mid-June to early July 2020), there was much discussion in the media about the way in which the possibility of support bubbles for people living alone represented a lifting of various “bans” that had effectively been (or had been perceived as being) in place since March. Such references to “bans” continued in public discourse throughout the year as guidance changed and restrictions were eased, then reimposed. In some cases, these references were reflective of the degree to which law and guidance had been blurred in government rhetoric; some things that were perceived as being illegal were legal, just against government guidance (Gayle, 2021). Tom Hickman (2020, p. 3) has argued that the latter in fact came to be constructed as “a powerful new, *sui generis* form of emergency regulatory intervention”; and a similar discussion of instances of “[d]issonance between official advice and underlying legal obligation” occurred in New Zealand (Knight, 2021, para.39).

In the context of the introduction of support bubbles in the United Kingdom, reference was repeatedly made to sex and hugging (Jones, 2020; Kelsey, 2020; Sini, 2020). That both had been restricted and indeed continued to be restricted or perceived as such, seemed to serve in public debate as a reminder of the extent to which everyday life had been regulated since March 2020. Formally, this regulation was set out in an ever-growing body of lockdown law, but its intense exceptionality, and the effects of its blurring with guidance, was most succinctly represented in other forms. For example, on several occasions over the course of the months of restrictions, the question of the legality of sitting on public benches was raised. During the first national lockdown in particular, instances of taped-over public benches came to symbolise both the regulation of everyday life and the impossibility of a break from a reality that had ruptured normality.

Other countries handled the question of sex differently. In the Netherlands, for example, the National Institute for Public Health and the Environment issued guidance following criticism of their “intelligent” lockdown, which permitted small gatherings providing that social distancing requirements were adhered to. This position ruled out the possibility of physical contact for people living alone (BBC, 2020b). The “[a]dvice on sexuality” recognised that: “It makes sense that as a single [person] you also want to have physical contact”. Initially, people were advised to “meet with the same person to have physical or sexual contact (for example, a cuddle buddy or ‘sex buddy’)” (BBC, 2020b). These terms were subsequently dropped in the light of the “commotion” they caused, but the basic message of making–and critically, being able to make—“good arrangements” to have sex was retained (Rijksinstituut voor Volksgezondheid en Milieu, 2020).

In the United Kingdom, the question was never dealt with in any comparable way. It was touched on lightly when the national lockdown was introduced, insofar as couples who lived apart were told that they needed to move in together or not meet up at all. Subsequently, it went unaddressed until support bubbles were introduced. Even then, it remained overlooked. For example, people living in house shares had to select one person living alone with whom the entire household would then bubble.

For those who were eligible to form support bubbles, the construct enabled both reconnections and new connections. Expanded bubbles in New Zealand and support bubbles in England, Scotland, and Wales were seemingly mostly used to reconnect with family members (Long et al., 2020, p. 33; Office for National Statistics, 2020), but newer connections developed too. Evidence from New Zealand pointed to the way in which some newer relationships developed, both through the lockdown “buddying” system and when the possibility of expanding household bubbles was introduced. Nicholas Long et al. (2020, p. 22) noted of their survey respondents that “[m]any buddying relationships seemed to have occurred by happenstance” and that when it came to expanded bubbles, respondents “did not necessarily fall back into pre-existing social relationships; they actively sought out those who needed their assistance and gladly provided it” (Long et al., 2020, p. 55). Anecdotal evidence from the United Kingdom highlighted the role that the possibility of forming a support bubble played in some new dating relationships (Found, 2021; Scott, 2021). As a legal construct, the support bubble was quite distinctive in this respect in drawing together relationships that would more commonly be portrayed in law as of different kinds, but were here united as relationships of support. It drew in, and built on, existing relationships, created a possibility for new relationships to develop, and provided a new way of being together.

The effect of the notion of the cross-household support bubble was not only on those who were eligible to form a bubble, nor even only on those with whom bubbles were formed or declined. It also affected those who were not eligible to form a bubble, and those who were eligible but nevertheless did not or could not do so, either at all, or with the person or people they wanted to. The kinds of anxieties that would, or could, need to be negotiated in navigating this new structure–and in forming, maintaining, and dissolving a bubble–became a point of discussion. This was not only where bubbles were formalised in law, but also where they were informally created, as in the US (Gutman, 2020; Weiner, 2020). Drawing on their research in New Zealand, Long et al. (2020, p. 55) highlighted the importance of communication and thinking through what being in or extending a bubble would involve, bearing in mind that while: “Expanding a bubble might feel like ‘reconnecting’ with loved ones … it is actually a new way of connecting with loved ones–the creation of a new social form”. The construct of the support bubble encapsulated and enabled certain and familiar ways of being together; it also set itself up as a new way of relating. Where it was formalised in law, as in New Zealand and the United Kingdom, it was constructed as a new legal form, implying that the question of the meaning of being together in a bubble was mediated in and through law.

## The Legal Construction of Relationships

From its inception, the concept of the bubble was inseparable from the assumptions made about the household unit to which it was attached. Households were constructed as largely bounded in this context, even though care and life are not so bounded. As Long (2020) subsequently argued in relation to the first national lockdown in the United Kingdom:

“the strictly bordered and individuated ‘households’ within which the United Kingdom government has sought to contain coronavirus are sociologically artificial, with confinement to such networks sometimes proving a source of great distress.” (Long, 2020, p. 253)

Jackie Gulland (2020, p. 336) also emphasised the way in which United Kingdom lockdown regulations had overlooked lived reality, arguing that they were structured by two visions: on the one hand, they were “constrained by assumptions that care happens either in the government, private and charitable care sectors or that it can be contained within a household”; on the other hand, they involved a “continuing focus … on households as autonomous, safe, adequate and secure, disguising the interdependency of human life, gendered aspects of caring and the inequalities of housing and living conditions”. In New Zealand, it was similarly argued by Susanna Trnka and Sharyn Davies (2021, pp. 168–171) that, although the exceptions to the original model of household confinement did go “a long way in recognizing that families do not necessarily map onto a single household”, the concept of the bubble itself “did not allow for the breadth and diversity of care relations that extend across multiple households”; nor did they “adequately address the needs of those who live alone or with others with whom they have little or no economic or social interconnection”.

In many respects, the lockdown laws in which the legal construct of the support bubble was situated–the laws relating to the home, personal movement, and familial and social life–reflected assumptions that are a structuring feature of the law and policy pertaining to close relationships more broadly. In the United Kingdom, these include assumptions about coupledom as a norm (Roseneil et al., 2020; Wilkinson, 2013) and the nuclear family (Brown, 2019). The concept of the cross-household bubble disrupted some of the associated assumptions that had underpinned earlier lockdown policy; for example, that “single people could exist in isolation from other households” (Gulland, 2020, pp. 332–333). It also revealed other base assumptions that underpinned modelling and policy in this context, such as about adherence, uptake, and engagement (Leng et al., 2021; Willem et al., 2021). More implicit assumptions were also included, such as that “people would automatically know what their bubbles would look like” and that those eligible to form cross-household bubbles had people in their lives with whom they could do so (Trnka and Davies, 2020). This latter assumption was similarly reflected in the “lockdown buddy” possibility introduced in New Zealand for people living alone. In their research, Long et al. (2020, pp. 21–22) noted that some of those who would have been eligible to pair up with a buddy in this way “used the 48-h notice period before Level 4 began to move in with loved ones so they could have company during lockdown”. Of those respondents who had lived alone during the Level 4 lockdown, however, only 18.6% had paired up with a buddy, and the most common reason given for not doing so was “not knowing another person living alone who lived close enough for them to legitimately buddy-up with”. For these respondents, the very assumption that underpinned the possibility of the lockdown buddy fell short of their reality.

At the same time as it reflected embedded assumptions about relationships, the legal concept of the support bubble also represented a distinctive development when looked at from the perspective of the legal recognition and regulation of close relationships. Firstly, the concept–especially in the United Kingdom context–enabled those who were eligible to define the meaning and nature of a support-bubble relationship. The framework within which this had to be done was tight, and it reflected a series of assumptions about needs and vulnerabilities, which needs counted, who had those needs, and the meaning of support itself. Those eligible nevertheless had a hypothetical degree of freedom with regards to the composition of their bubble. The intention was that bubbles would be formed with family members, partners, loved ones, or friends, and it seems that, mostly, they were (Office for National Statistics, 2020), but this was not a requirement.

Secondly, and relatedly, through the concept of the support bubble, some kinds of relationship that had not necessarily attracted much previous legal attention–like friendships and dating relationships–came to find a space in which they were accorded a degree of legal reflection and recognition. A rich literature argues that family law, which is primarily oriented around the “sexual family” and the structures of marriage and parenthood (Fineman, 1995; Hasday, 2014), should concern itself more with practices of care and relationships that it has overlooked, such as friendships (Herring, 2015; Westwood, 2013). As a new legal form that was exclusively focused on a certain idea of support itself, the concept of the support bubble thus presented an interesting case. However, it also revealed, as did lockdown laws more generally, the effects that law has on the lived reality of relationships. The concept of the bubble enabled some ways of being together and excluded others; more specifically, it took ways of being and recast these in a new socio-legal form.

## Discussion and Conclusion

Where it was introduced, the concept of the support bubble presented a distinctive way of thinking about, relating to, and being with one another in a previously unthinkable “time of pandemic” (Lear, 2021, pp. 3–5). This article has given a sense of the level and complexity of intervention in personal, familial, and social life that occurred in this period in New Zealand and the United Kingdom. In this context, the concept of the bubble drew in and built on existing relationships, created a possibility for new relationships to develop, and was itself a new way of being together. Where support bubbles were formalised as a matter of law, as in the cases analysed, the meaning of being together in this way was, in part, mediated in and through law: a form of relating was legally constructed and real relationships were affected through law.

This observation raises the question of the longer-term implications of the concept of the support bubble itself. If COVID-19 has “changed the way in which we look at ourselves and others in many ways, and … our relationship with the world and our sense of what we value in it” (De Rosa and Mannarini, 2021, p. 9), then what of the concept of the bubble? This article has suggested that, particularly in its cross-household form, the concept carried a disruptive potential for the legal regulation of close relationships. This is because of the way in which it brought together relationships that would not ordinarily have been categorised together or treated as comparable as a matter of law, but also because it created some space for relationships that are not ordinarily accorded much legal recognition, such as friendships. At the same time, the concept of the bubble served as a reminder of the disruptive potential of the law itself for the lived reality of these same relationships. It constituted a new legal form, and one that reflected a fundamental point: that of the many ways in which law constructs and acts on ways of thinking about, relating to, and being with one another.
